# Pupillometry: Psychology, Physiology, and Function

**DOI:** 10.5334/joc.18

**Published:** 2018-02-21

**Authors:** Sebastiaan Mathôt

**Affiliations:** 1 Rijksuniversiteit Groningen, NL

**Keywords:** pupillometry, pupil light response, pupil near response, psychosensory pupil response, orienting response, eye movements

## Abstract

Pupils respond to three distinct kinds of stimuli: they constrict in response to brightness (the pupil light response), constrict in response to near fixation (the pupil near response), and dilate in response to increases in arousal and mental effort, either triggered by an external stimulus or spontaneously. In this review, I describe these three pupil responses, how they are related to high-level cognition, and the neural pathways that control them. I also discuss the functional relevance of pupil responses, that is, how pupil responses help us to better see the world. Although pupil responses likely serve many functions, not all of which are fully understood, one important function is to optimize vision either for acuity (small pupils see sharper) and depth of field (small pupils see sharply at a wider range of distances), or for sensitivity (large pupils are better able to detect faint stimuli); that is, pupils change their size to optimize vision for a particular situation. In many ways, pupil responses are similar to other eye movements, such as saccades and smooth pursuit: like these other eye movements, pupil responses have properties of both reflexive and voluntary action, and are part of active visual exploration.

Seeing is an activity. We do not passively let visual information fall onto our retina, but actively seek out objects of interest by moving our body, head, and eyes. The saccadic and smooth-pursuit eye movements that control gaze direction have been extensively studied (e.g. Kowler, 2011). But eye movements do far more than direct gaze. Once gaze has been directed at an object of interest, our eyes continue to move to provide our brain with the best possible image: the curvature of the lens changes (*accomodates*) to control focus; and our pupils enlarge (*dilate*) or shrink (*constrict*) to control how much of the lens’s surface is exposed, and consequently how much light enters the eye. In this review, I will focus on this last type of eye movement: pupil responses.

The pupil changes its size in response to three distinct kinds of stimuli: it constricts in response to brightness (the *pupil light response*, or PLR) and near fixation (the *pupil near response*, or PNR); and it dilates in response to increased cognitive activity, such as increased levels of arousal or mental effort (the *psychosensory pupil response*, or PPR).

Pupil responses are partly reflexive, in the sense that the same stimulus always leads to a qualitatively similar response: pupils always constrict, and never dilate, in response to light. But pupil responses are also partly voluntary, in the sense that they are modulated by high-level cognition: when you choose to attend to a light in peripheral vision, your pupils constrict more than when you choose to ignore this light (e.g. Binda & Murray, 2015). This is similar to other eye movements, such as saccades and smooth pursuit, which also have properties of both reflexive and voluntary action (e.g. Zoest, Stigchel, & Donk, 2017). One aim of this review is to discuss how the different kinds of pupil responses are modulated by high-level cognition.

Current understanding of pupil responses is largely descriptive. We know what kind of stimuli trigger pupil responses, and we know, more or less, which neural pathways underly these responses. But we do not fully understand *why* the pupil responds in the way it does. This is different from other eye movements, for which the function is generally clearer; for example, saccadic and smooth pursuit eye movements serve to stabilize the retinal image, and to bring relevant objects into foveal vision (Land, 1999; Walls, 1962)—that is fairly clear. But why do your pupils dilate when you get aroused? Another aim of this review is to consider this crucial question: how do pupil responses help you to better see the world?

## Anatomy and neural pathways

### Anatomy of the eye

**Figure 1:**
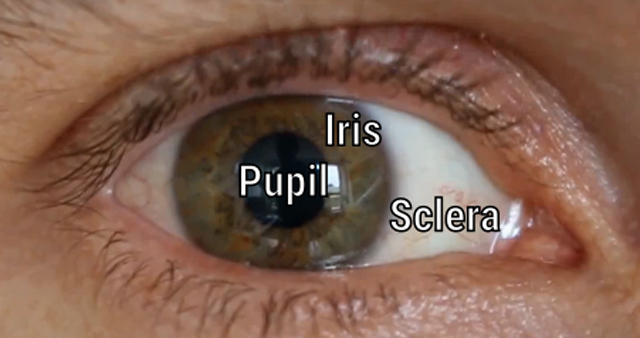
A photo of my own eye, showing the pupil, iris, and sclera.

**Figure 2:**
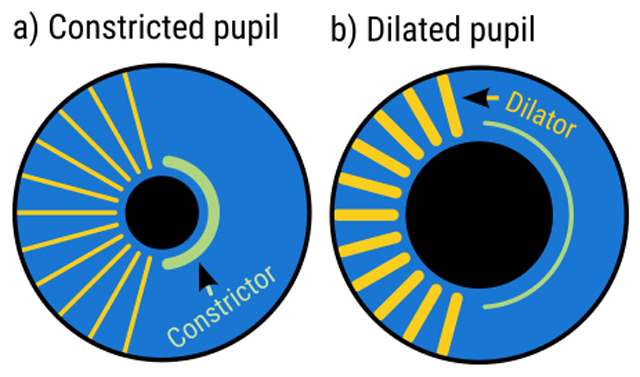
**a)** When the iris sphincter muscle (green) contracts, it tightens the inner side of the iris, thus causing the pupil to constrict. **b)** When the iris dilator muscle (yellow) contracts, it pulls the inner side of the iris outward, thus causing the pupil to dilate.

The *pupil* is a transparent opening in the center of the eye (Figure 1). Light passes through the surface of the lens that is exposed by the pupil, and is focused onto the retina in the back of the eye. The pupil normally appears black, because the inside of the eye is dark, and not because the pupil is an opaque black surface. (This becomes apparent when a camera flash illuminates the inside of the eye, which under a certain angle makes the pupil appear red.) The diameter of the human pupil varies between roughly 2 and 8 mm; therefore, the pupil can change the amount of light that enters the eye by a factor of roughly 16. The colored area around the pupil is the *iris*, and it contains the muscles that control pupil size (Figure 2). The white tissue around the iris is called the *sclera*. The transparent tissue that covers the iris and the pupil (but not the sclera) is the *cornea*.

### Neural pathways

Pupil size is controlled by two pathways that, although interconnected, are often considered distinct: the *parasympathetic constriction pathway* and the *sympathetic dilation pathway*. Different reviews highlight different aspects of these pathways. Below I focus on those areas and connections for which several authoritative reviews agree that they are important for pupil control (see Kardon, 2005; McDougal & Gamlin, 2008; Samuels & Szabadi, 2008; Szabadi, 2012; C. Wang & Munoz, 2015). The information below is adapted from these reviews, and detailed references can be found there, especially in Kardon (2005) and McDougal & Gamlin (2008).

#### The constriction pathway

**Figure 3:**
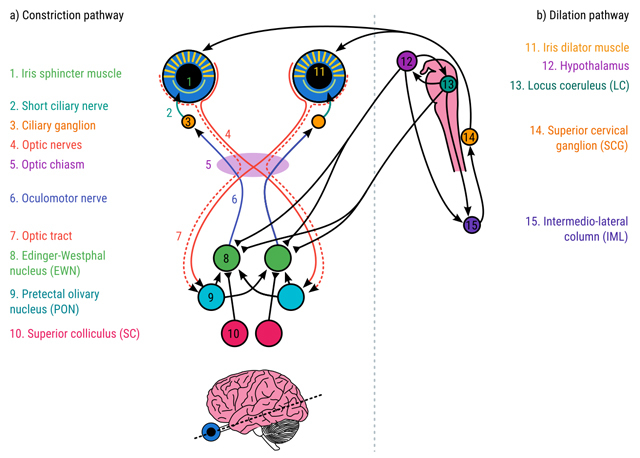
**a)** The pupil constriction pathway. **b)** the pupil dilation pathway. (Based on Kardon, 2005; McDougal & Gamlin, 2008; Samuels & Szabadi, 2008; Szabadi, 2012; C. Wang & Munoz, 2015).

Pupil constriction is controlled by the *iris sphincter muscle*. The iris sphincter muscle encircles the pupil like a cord that reduces the size of the pupil when it contracts (Figure 2a). The iris sphincter is innervated by the parasympathetic nervous system, the part of the autonomic nervous system that is involved in homeostasis (i.e. keeping the body in stable condition); the link between pupil constriction and the parasympathetic nervous explains why pupils are relatively small at rest. The constriction pathway is a subcortical pathway that connects the retina to the iris sphincter muscle (Figure 3a).

The constriction pathway as described here corresponds to those areas that are commonly considered crucial for pupil constriction, and is essentially that of the pupil light response (PLR). Notably absent is the lateral geniculate nucleus (LGN) of the thalamus, which, in many unreferenced descriptions that can be found online, sits between the retina and the EWN. However, more authoritative reviews (e.g. Kardon, 2005) assume that the retina-geniculate pathway is only involved in vision, and not in pupil responses.

- As light falls on the retina, nerve impulses are sent along the optic nerve to the optic chiasma.
- The optic chiasma combines input from the retina in both eyes, re-organizes it based on visual field, and sends it to the pretectal nucleus (PN); that is, information from the left visual field goes to the PN in the right hemisphere, and the right visual field goes to the left PN.
- From the PN, information is sent to the Edinger-Westphal nucleus (EWN). Each EWN receives input from the left and right PN, thus combining information from the left and right visual fields; that is, both the left and right EWN receive input from both eyes and both visual fields.
- From the EWN, information is sent via the Oculomotor Nerve (III) to the ciliary ganglion (CG), which is located just behind the eye.
- From the CG, information is sent via the short ciliary nerves to the iris sphincter muscle.

#### The dilation pathway

Pupil dilation is controlled by the *iris dilator muscle*. The dilator muscle consists of fibers that are oriented radially, and connect the exterior of the iris with the interior. When the dilator muscle contracts, it pulls the interior of the iris outward, thus increasing the size of the pupil (Figure 2b). The iris dilator muscle is controlled by the sympathetic nervous system, the part of the autonomic nervous system that is involved in arousal, wakefulness, and the fight-or-flight response; the link between pupil dilation and the sympathetic nervous system explains why pupils are relatively large when someone is aroused. The dilation pathway is a subcortical pathway that starts at the hypothalamus and the locus coeruleus (LC) and connects to the iris dilator muscle (Figure 3b).

- The LC is especially active when an organism is aroused, awake, and alert; that is, the LC reflects arousal. (But see the section on the Adaptive Gain theory for a more nuanced discussion of LC function.) The LC projects to the intermedio-lateral column (IML) of the spinal cord.
- The hypothalamus is a complicated structure with many connections and subnuclei; however, in the context of pupil dilation its role is similar to that of the LC: activity in the hypothalamus reflects arousal and wakefulness, and it projects to the IML. The hypothalamus and LC also have reciprocal excitatory connections.
- The IML projects to the superior cervical ganglion (SCG), located just outside the spinal cord.
- The SCG projects, via a complicated network of nerves, to the iris dilator muscle.

The neural pathway that leads to pupil dilation is understood less well than that leading to constriction, especially because it involves brain areas, such as the LC and hypothalamus, that are involved in many aspects of cognition. However, the pathway described here corresponds to those areas that are commonly considered to be crucial.

#### Interactions between the constriction and dilation pathways

Although the constriction and dilation pathways are roughly distinct, they interact in at least three ways:

- The LC inhibits the EWN; that is, LC activity causes pupil dilation not only by activating the sympathetic dilation pathway, but also by inhibiting the parasympathetic constriction pathway at the level of the EWN. It has even been suggested that this is the primary pathway that underlies pupil dilation in response to arousal and mental effort (Steinhauer, Siegle, Condray, & Pless, 2004).
- Similarly, the intermediate layers of the superior colliculus (iSC), which are traditionally not considered part of the pupil pathways, inhibit the EWN; presumably, these inhibitory connections drive the rapid pupil dilation that accompanies the orienting response (C. Wang & Munoz, 2015).
- The effect of light is twofold. First, and most directly, light activates the constriction pathway, causing the pupil to constrict. But light also induces wakefulness, and activates the dilation pathway, via a connection through the suprachiasmatic nucleus (SCN), which is part of the hypothalamus, and the dorsomedial hypothalamus (DMH) to the LC. In other words, light drives pupil constriction through a direct pathway, and pupil dilation through an indirect pathway.

## The pupil light response

### Profile

**Figure 4:**
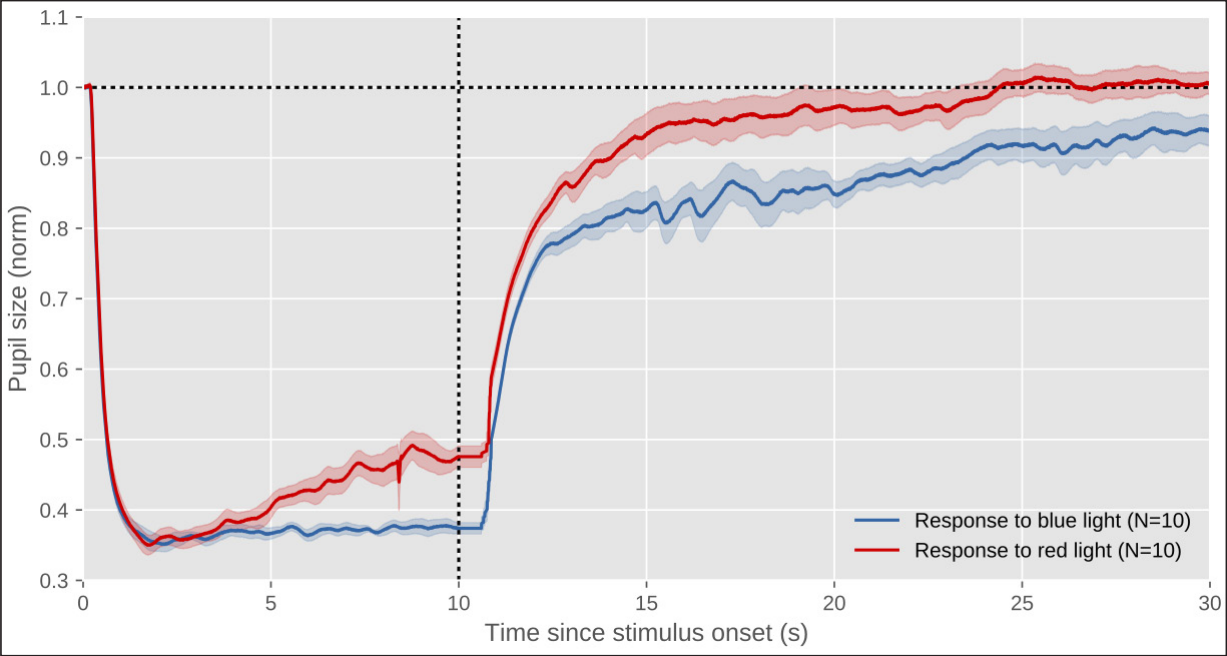
The profile of a typical pupil light response. This figure shows data of myself while I’m looking at red or blue full-screen colors on a computer display (N = 10 trials per color). The x axis indicates time since stimulus onset. The y axis indicates pupil size as a proportion of pre-stimulus pupil size. Errors bars reflect the standard error. All data shown in this figure and others is available through the URL provided at the end of the article.

The pupil light response (PLR), also called the pupil light reflex, is the constriction of the pupil in response to brightness, and the dilation of the pupil in response to darkness. Figure 4 shows a typical PLR, elicited by 10 s of blue or red light presented on a computer monitor, followed by 20 s of a dark screen.

The profile of a typical PLR looks as follows:

- [Light on] 0–0.2s: This is the latency period during which the pupil does not yet respond. The exact latency depends on many factors, such as stimulus intensity (latencies decrease with stimulus intensity) and age (latencies increase with age) (Ellis, 1981).
- [Light on] 0.2–1.5s: The pupil constricts strongly and rapidly until it reaches its minimum size.
- [Light on] 1.5–10s: The pupil either remains fully constricted while the light remains on, or unconstricts (redilates) slightly. This unconstriction, when it occurs, is sometimes called *pupil escape*; whether it occurs depends on the color of the light: blue light leads to sustained constriction, whereas red light leads to pupil escape. This difference results from the different photoreceptors that are sensitive to blue and red light, as described below.
- [Light off] 10 s–30s: The pupil gradually recovers to its original size. Dilation due to light offset occurs much more slowly than constriction due to light onset. It can take many seconds for the pupil to fully recover, and recovery is faster for red than blue light, again because of the photoreceptors that are sensitive to red and blue light. After a high-intensity blue light, the pupil remains slightly constricted for many minutes. This is called the post-illumination pupil response (PIPR) (Markwell, Feigl, & Zele, 2010).

### Neural basis and photoreceptors

The PLR is driven by all known types of photoreceptors: rods, cones, and intrinsically photosensitive retinal ganglion cells (ipRGCs) (Kardon, 2005; Markwell et al., 2010; McDougal & Gamlin, 2008).

Cones are sensitive to color, in the sense that there are three types of cones that are maximally responsive to different colors, and the relative activation of these different cone types allows us to distinguish between different colors. (That is, individual cones do not ‘see color’). Cone density is highest in the fovea, and (compared to rods) cones require intense light to become active; therefore, cones dominate central vision, and vision in medium-to-bright levels of light.

Rods are not sensitive to color, in the sense that all rods are maximally sensitive to the same shade of blueish green, and can therefore not distinguish between different colors. Rods are mostly absent from the fovea, and (compared to cones) respond also to weak light; therefore, rods dominate peripheral vision in darkness.

ipRGCs are ganglion cells that receive input from rods and cones, but also contain a photopigment (melanopsin) themselves (Berson, Dunn, & Takao, 2002). ipRGCs respond much more slowly to light than rods and cones do. In addition to their role in the PLR (as described below), ipRGCs project to the suprachiasmatic nucleus (SCN) of the hypothalamus (sometimes called the biological clock) to maintain the circadian (day-night) rhythm. ipRGCs respond maximally to blueish light (Markwell et al., 2010). ipRGCs seem to contribute mostly to non-image-forming vision, that is, visual functions that are not accompanied by conscious visual awareness, such as pupil responses and maintenance of circadian rhythm. (That is, we are not consciously aware of the effect that light has on synchronizing our circadian rhythm.) However, some studies suggest that ipRGCs may also play a minor role in conscious visual perception (Ecker et al., 2010; Zaidi et al., 2007).

Rods and cones drive the initial pupil constriction of the PLR (0.2–1.5s). This initial constriction therefore has many of the same properties as regular human vision, including that it is strongest for light presented in central vision (Crawford, 1936; Hong, Narkiewicz, & Kardon, 2001).

However, input from rods and cones desensitizes quickly; therefore, if the PLR was based only on rod and cone vision, the pupil would rapidly unconstrict even while the light was still on. ipRGCs are the reason why our pupils stay constricted: once rod and cone input reduces, ipRGCs start responding and keep the pupil constricted for as long as the light is on (Gamlin et al., 2007). Figure 4 shows indirect evidence for the role of ipRGCs in maintaining pupillary constriction: given an equally strong initial constriction, pupil constriction (1.5–10s) is more sustained for blue than red light, because ipRGCs are maximally sensitive to blueish light. ipRGCs also drive the PIPR, which is therefore also strongest for blue light. Simply put: because of rods and cones, the pupil rapidly constricts in response to sudden light increases; but because of ipRGCs, the pupil stays constricted throughout the day (Gooley et al., 2012; Wong, 2012).

Signals from rods, cones, and ipRGCs go to the pretectal olivary nucleus (PON), and from there follow the pupillary constriction pathway as shown in Figure 3a.

### Cognitive influences

Historically, the PLR was considered a purely reflexive response to the amount of light that falls on the retina. However, recent studies—and also some recently rediscovered older studies—have shown that the PLR is not merely a reflex, but is affected by how visual input is selected (i.e. visual attention), processed, and interpreted (for reviews, see Binda & Murray, 2014; Mathôt & Van der Stigchel, 2015).

Whereas the PLR is a large response (see Figure 4), cognitive influences on the PLR are generally modest in size. For example, Binda, Pereverzeva, & Murray (2013a) reported that covertly attending (without making an eye movement) to a bright stimulus triggered a pupil constriction that was about three times weaker than that triggered by directly looking at a bright stimulus. In most other studies, cognitive influences on the PLR are not directly contrasted with direct exposure to brightness or darkness; but cognitive influences on the PLR are generally small, corresponding to a pupil-size change of less than 1% (Bombeke, Duthoo, Mueller, Hopf, & Boehler, 2016) to about 5% (Mathôt, van der Linden, Grainger, & Vitu, 2013).

#### Visual awareness

Several elegant studies using binocular rivalry, dating back almost a century, already revealed cognitive influeces on the pupil light response (Bárány & Halldén, 1948; Brenner, Charles, & Flynn, 1969; Harms, 1937; Lowe & Ogle, 1966; for recent replications, see Fahle, Stemmler, & Spang, 2011; Kimura, Abe, & Goryo, 2014; Naber, Frassle, & Einhauser, 2011). For example, Bárány & Halldén (1948) presented a horizontal line to one eye, and a vertical line to the other eye. This different visual input to both eyes induced binocular rivalry: participants sometimes consciously perceived the horizontal line, and sometimes the vertical line, but rarely both. In addition, the researchers occasionally flashed a dim light into one of the eyes, and recorded whether this flash triggered a measurable pupil constriction or not. Crucially, they found that a measurable pupil constriction was more likely to be triggered when the light was flashed into the eye that, at that moment, dominated visual awareness. Phrased differently, the PLR was strongest for light sources that were consciously perceived—a clear demonstration of how high-level cognition influences the PLR.

#### Covert visual attention

More recently, it has been shown that covert visual attention also modulates the PLR (Binda & Murray, 2015; Binda et al., 2013a; Binda, Pereverzeva, & Murray, 2014; Bombeke et al., 2016; Ebitz, Pearson, & Platt, 2014; Mathôt, Dalmaijer, Grainger, & Van der Stigchel, 2014a; Mathôt et al., 2013; Naber, Alvarez, & Nakayama, 2013; Olmos-Solis, van Loon, & Olivers, 2018; Unsworth & Robison, 2016). For example, in one of our own experiments, participants fixated their gaze in the center of a display that was bright on one side, and dark on the other side (Mathôt et al., 2013). We then presented a cue (such as a voice saying “left” or “right”) that indicated the probable location of an upcoming target. Without moving their eyes, participants shifted their attention to the cued side, which could be either dark or bright. Crucially, we found that pupils were smaller when participants covertly attended to the bright side of the display, compared to the dark side of the display. In other words, covertly attending to something that is bright or dark causes a PLR, just like (although much more weakly than) directly looking at something that is bright or dark. This effect is so robust that it can even be used to decode whether people are covertly attending to something bright or dark, with around 90% accuracy on a single-trial level (Mathôt, Melmi, van der Linden, & Van der Stigchel, 2016).

Neurophysiological studies have shown similar effects (Ebitz & Moore, 2017; C. Wang & Munoz, 2016). For example, Ebitz & Moore (2017) stimulated neurons in the frontal eye field (FEF) of the rhesus macaque. The FEF is a brain area in the prefrontal cortex (PFC), and is involved in eye movements and covert shifts of attention: strong (suprathreshold) FEF stimulation triggers eye movements; weak (subthreshold) stimulation triggers covert shifts of attention. In their study, Ebitz & Moore (2017) first stimulated FEF neurons to trigger a covert of shift of attention to the receptive field (RF) of these neurons (i.e. the location in the visual field to which these neurons respond); next, they flashed a stimulus either within, or outside of, the stimulated RF. Crucially, they observed a stronger PLR to stimuli flashed within the stimulated RF, compared to outside of the stimulated RF—a result that echoes behavioral studies (notably Binda & Murray, 2015; Olmos-Solis et al., 2018).

#### Eye-movement preparation

When you prepare an eye movement toward an object, but before your eyes actually set in motion, you already begin to perceive the to-be-looked-at object more clearly in your mind’s eye; phrased differently, each saccadic eye movement is preceded by a covert shift of attention (Deubel & Schneider, 1996; Hoffman & Subramaniam, 1995; Kowler, Anderson, Dosher, & Blaser, 1995). Given the results discussed above, a natural question is whether this presaccadic shift of attention results in a preparatory pupil light response; that is, when you make an eye movement toward a lamp, does your pupil already begin to constrict before your eyes start to move?

To test this, we conducted an experiment in which participants initially fixated in the center of a display that was divided into a bright and a dark half (Mathôt, van der Linden, Grainger, & Vitu, 2015b; see also Ebitz et al., 2014). Next, an auditory cue (again a voice saying “left” or “right”) instructed participants to make an eye movement toward either the left or the right side of the display, which could be either bright or dark. Crucially, we found that the pupil began to respond (weakly) to the brightness of the cued side already while the eyes were still in motion. Because the minimum latency of the PLR is about 200 ms (Ellis, 1981, see also Figure 4), this zero-latency response clearly resulted from preparation, presumably mediated through a presaccadic shift of attention.

That a PLR is prepared along with an eye movement, rather than being a passive consequence of the brightness of a newly fixated location, makes sense given the tight coupling between attention and eye movements (Deubel & Schneider, 1996; Hoffman & Subramaniam, 1995; Kowler et al., 1995). And it also makes ecological sense, considering that humans make about three eye movements per second (e.g. Rayner, 1998). Given that it takes at least 200 ms for the pupil to respond to light (Ellis, 1981), this would mean that by the time that the pupil start to respond to the brightness of a newly fixated object, gaze has already shifted elsewhere; or at least that’s what would happen in the absence of any preparation. By reducing the effective latency of the PLR, preparation may allow the sluggish PLR to keep up with our eye movements.

#### Subjective interpretation

Whether a stimulus is subjectively interpreted as being bright (or not) also determines how strongly the pupil responds to it (Binda, Pereverzeva, & Murray, 2013b; Bombeke et al., 2016; Laeng & Endestad, 2012; Naber & Nakayama, 2013; Zavagno, Tommasi, & Laeng, 2017). For example, Naber & Nakayama (2013) showed images, either photographs or cartoons, to participants. Crucially, they found that images that contained a sun triggered stronger pupil constriction than images that did not contain a sun, even when eye position and objective luminance were controlled for. This effect disappeared when images were flipped vertically, presumably because vertically flipped images are difficult to interpret.

From this finding, the authors concluded that the PLR reflects subjective interpretation: an image that we interpret as being very bright (such as an image of the sun) causes a strong pupil constriction, even when the objective luminance of the image is modest.

#### Mental imagery and word meaning

In the studies described above, pupil responses were always triggered by visual stimuli (even though the strength of the pupil response was modulated by cognitive influences). However, several studies suggest that the pupil may even constrict in response to mental images, that is, in response to an internally generated image without actual visual input (Laeng & Sulutvedt, 2014; Mathôt, Grainger, & Strijkers, 2017).

In one experiment, Laeng & Sulutvedt (2014) asked participants to look at a uniformly gray screen, while mentally picturing that they were in a particular environment. Crucially, Laeng & Sulutvedt (2014) found that participants’ pupils constricted when they mentally pictured being in a bright, compared to a dark, environment; that is, the pupil was smaller when participants imagined themselves outdoors under a sunny sky, compared to indoors in a dark room.

In a series of our own experiments, participants read or heard individual words (Mathôt et al., 2017). To make sure that participants processed the meaning of the words, they were instructed to press spacebar whenever the word was an animal name. Some of the non-animal words were associated with brightness (e.g. ‘sun’); others were associated with darkness (e.g. ‘night’). Crucially, we found that participants’ pupils were smaller when they read or heard words that conveyed a sense of brightness, compared to words that conveyed a sense of darkness. Echoing the study of Laeng & Sulutvedt (2014), we interpreted this in terms of mental imagery: when participants read a word, they automatically simulate the perceptual properties of the word’s referent (e.g. Pulvermüller, 2013; but see Mahon & Caramazza, 2008 for a different perspective); for example, when participants read ‘sun’, they automatically create a mental image of a bright ball of fire in the sky. And, plausibly, this mental image then causes the pupil to constrict.

#### Working memory

When discussing the PLR in the context of visual working memory, it is important to clearly distinguish this from the classic studies of Kahneman & Beatty (1966) and others. Kahneman & Beatty (1966) have shown that the pupil dilates when you keep items in working memory; this is an effect of mental effort, which we will get back to below in the section on the psychosensory pupil response. In contrast, the studies described in the current section address a different question: can we tell *what* someone is keeping in memory by looking at pupil size? More specifically, can we use the PLR to track whether someone keeps something bright or something dark in working memory?

In one of our experiments, participants memorized a location, which could be either on a dark or a bright background (Fabius, Mathôt, Schut, Nijboer, & Van der Stigchel, 2017; see also Unsworth & Robison, 2016). We found that the pupil was smaller when participants memorized a location on a bright, compared to a dark, background. This suggests that memorizing a location is similar to covertly attending to a location, in line with previous research (e.g. Awh, Jonides, & Reuter-Lorenz, 1998). However, in this study, the bright/dark background was continuously visible; therefore, the effect was not necessarily (and was probably not) driven by a memory representation of brightness or darkness per se, but rather by a more-or-less sustained shift of attention to the bright or dark background. In other words, the effect of spatial working memory on the PLR is presumably mediated by covert visual attention.

Olmos-Solis et al. (2018) used a different approach to probe visual working memory through pupillometry. In their study, participants memorized a color, which defined the target stimulus during a subsequent visual-search task. In the retention interval before the visual-search task, an irrelevant probe was briefly presented. Crucially, the color of this probe either matched, or did not match, the memorized color. Olmos-Solis et al. (2018) found that the pupil constricted more in response to memory-matching as compared to non-matching probes; in other words, when a stimulus matches the contents of visual working memory, it triggers a stronger PLR. This finding is again mediated by visual attention; more specifically, it is mediated by the fact that stimuli that match the contents of visual working memory tend to capture attention (e.g. Olivers, Meijer, & Theeuwes, 2006).

To test whether a memory representation of brightness or darkness also triggers a PLR, we conducted another series of experiments in which we asked participants to memorize either bright or dark stimuli (Blom, Mathôt, Olivers, & Van der Stigchel, 2016); that is, rather than asking participants to memorize a location on a continuously visible bright or dark background (Fabius et al., 2017) or measuring the pupil response to a probe (Olmos-Solis et al., 2018), we asked participants to memorize bright or dark objects that were subsequently removed from the display, such that any differences in pupil size would necessarily be driven by the memory trace. However—and somewhat to our own surprise—we found no evidence that, after the stimuli had been removed from the display, maintaining bright or dark stimuli in working memory affected pupil size.

Given the studies described in the previous section (Mental imagery and word meaning), which showed that pupil size is affected by a mental image of brightness or darkness (Laeng & Sulutvedt, 2014), the finding that pupil size is *not* affected by keeping bright or dark objects in working memory is puzzling: Surely visual working memory is closely related to mental imagery? This apparent discrepancy illustrates that we do not yet fully understand how high-level cognition in general, and mental imagery in particular, modulate the PLR.

### Function

How does the PLR help visual perception? This seemingly obvious question does not have a clear-cut answer, and even experts often provide different, though not mutually exclusive, answers (Woodhouse & Campbell, 1975). But several functions are generally assumed to play a role (reviewed in Mathôt & Van der Stigchel, 2015).

The benefit of a dilated pupil is clear: A large pupil lets a lot of light into the eye, thus increasing the amount of visual information that the brain receives, thus increasing visual sensitivity; therefore, a dilated pupil, compared to a constricted pupil, allows you to better detect faint stimuli. Imagine a cat in the dark, looking for small moving stimuli (mice); this cat would probably benefit from large pupils that capture as much light as possible. The question thus becomes why the pupil is not always maximally dilated: why don’t the eyes always capture as much light as they can?

One answer that may come to mind is that pupil constriction serves to protect the retina from damage due to overexposure. However, there is little evidence that the range of light intensities that we normally encounter can damage the retina, even with a fully dilated pupil (Woodhouse & Campbell, 1975). And when you look directly at the sun (which can damage the retina), retinal light flux is so intense that a constricted pupil offers little protection. But if the PLR does not (primarily) serve to protect the retina from overexposure, then what is it good for?

First, a mobile pupil acts as a buffer when transitioning from brightness to darkness (Barlow, 1972; Woodhouse & Campbell, 1975). When you are in brightness, rods and cones become light adapted (‘bleached’), which makes them less sensitive to light. When you then go from brightness into darkness, rods and cones need to adapt. Dark adaptation is a gradual process that, especially for rods, takes tens of minutes. During the period that you are in darkness while dark adaptation is not yet complete, vision is impaired. Because the pupil can dilate much more rapidly than rods and cones can adapt, pupil dilation reduces the amount of dark adaptation that is necessary, thus (compared to a static pupil) improving vision during the first few minutes of dark adaptation. (Light adaptation is a very rapid process, and does not appear to benefit as much, or at all, from the PLR).

Second—and related to the pupil near response that will be discussed in the next section—pupil constriction increases depth of field: the range of distances at which vision is sharp (Campbell, 1957; Charman & Whitefoot, 1977). This is because a constricted pupil exposes only a small part of the eye’s lens. A small lens, compared to a large lens, suffers less from optical distortions that reduce depth of field. While focusing on a point that is very far away, a fully constricted pupil (±2 mm diameter) provides sharp vision from about 2 m distance to infinity, whereas a fully dilated pupil (±8 mm diameter) provides sharp vision from about 7 m distance to infinity. In most laboratory settings, stimuli are presented on a computer display at a fixed distance, and depth of field is therefore of little importance. However, the real world is three dimensional and contains many relevant objects at many different distances; therefore, in real life, the increased depth of field offered by a constricted pupil may be very important.

Third, pupil constriction increases visual acuity: how well you can discriminate fine detail (Campbell & Gregory, 1960; Liang & Williams, 1997; Woodhouse, 1975). Analogous to depth of field, this is because a constricted pupil, and thus a small lens, suffers less from optical distortions (spherical and chromatic abberations) that cause optical blur. Woodhouse (1975) estimated that a constricted pupil, compared to a dilated pupil, can improve visual acuity by about 20% in situations in which visual sensitivity is not a limiting factor (i.e. for discrimination of bright, high-contrast gratings). When the pupil becomes too small, a different set of optical distortions (diffractions) emerge that impair, rather than improve, visual acuity; this may be why the minimum size of the human pupil is about 2 mm, just above the size at which these distortions come into play.

The benefits of pupil constriction on visual acuity and depth of field are often described as two separate phenomena, but they are really two sides of the same coin: when the pupil constricts, focus improves for all objects except those that are already in perfect focus. For poorly focused objects, such as objects that are far beyond the point of fixation, the benefit is especially pronounced; this is why pupil constriction leads to a pronounced increase in depth of field. For objects that are fixated and are therefore already in good focus, the benefit is more subtle; therefore, pupil constriction leads to only a subtle increase in visual acuity at fixation. However, focus is never perfect: there are always slight imperfections in the eye’s lens, and different colors have slightly different focal depths, making perfect focus impossible even in theory. Therefore, pupil constriction always improves visual acuity, although the effect can be very small.

The PLR thus likely helps vision by reducing dark adaption, and by optimizing the trade-off between visual acuity and depth of field (which benefit from a small pupil) and visual sensitivity (which benefits from a large pupil, especially in darkness). In humans, these effects seem to be small, and vision would likely not be strongly impaired if the pupil would not respond to light. (Although this is difficult to prove, because there seems to be no naturally occurring condition in which pupil size is fixed while vision is otherwise healthy.) However, other animals have far larger ranges of pupil sizes than humans, and thus may benefit more from the PLR. For example, the area of the slit pupil of the Gecko can change by a factor of 300 (Denton, 1956).

And, even if in some animals (such as humans) the PLR may confer but little advantage, “one should bear in mind that seemingly trivial advantages may become extremely significant in a competitive situation; thus an animal whose pupil can dilate a little more may acquire a distinct competitive edge at dusk, and this could make the difference between starvation and plenty.” (Barlow, 1972, p. 3).

## The pupil near response

### Profile

**Figure 5:**
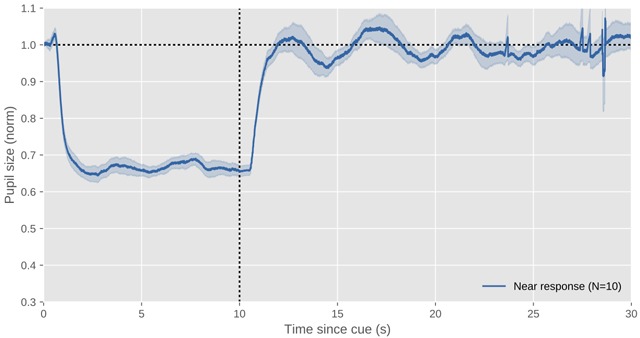
The profile of a typical pupil near response. This figure shows data of myself while I’m shifting gaze from a far-away to a nearby point (prompted by an auditory cue at 0 s) and back again (prompted by another auditory cue at 10 s; N = 10 trials). The x axis indicates time since the onset of the auditory cue to shift gaze. The y axis indicates pupil size as a proportion of pre-stimulus pupil size. Errors bars reflect the standard error. All data shown in this figure and others is available through the URL provided at the end of the article.

The pupil near response (PNR), also called the pupil near reflex, is the constriction of the pupil in response to looking at a nearby object, and the dilation of the pupil in response looking at a far-away object. The PNR is certainly the least studied, and perhaps the least understood of all pupil responses. The profile of the PNR, shown in Figure 5, is similar to that of the pupil light response (PLR).

In the example data, again from myself, shown in Figure 5, the PNR is elicited by an auditory cue at 0 s to shift gaze from far (±2 m) to near (±0.1 m), and a second auditory cue at 10 s to shift back from near to far. This shift of gaze was such that, except for vergence eye movements, eye position did not change. The profile of the resulting PNR looks as follows:

- [Far to near] 0–0.6s: This is the latency period of the PNR, relative to the cue onset. This time comprises both the time it takes to process the cue and to shift focus from far to near, and the latency of the PNR proper.
- [Far to near] 0.6s–2s: The pupil constricts strongly until it reaches its minimum size.
- [Far to near] 2s–10s: The pupil remains fully constricted. There is no notable pupil escape in the PNR.
- [Near to far] 10s–12s: The pupil recovers to its original size. This redilation is slower than the constriction; however, it is faster than the redilation after a light response (see Figure 4).

### The near triad

The PNR is part of three eye movements, the near triad, that usually (but not necessarily, e.g. Stakenburg, 1991) occur together (Mays & Gamlin, 1995; McDougal & Gamlin, 2008). Besides the PNR, the near triad includes: *vergence*, the inward rotation of the eyes to look at something nearby and the outward rotation of the eyes to look at something far away; and *accomodation*, the curving of the eye’s lens to focus on a nearby object and the flattening of the lens to focus on a far-away object.

### Neural basis

The output pathway of the PNR is the same as that of the PLR, projecting from the Edinger-Westphal nucleus (EWN) to the iris sphincter muscle (see Figure 3a). However, unlike the PLR, the PNR does not appear to be driven directly by a subcortical pathway, but rather by cortical projections to the EWN (McDougal & Gamlin, 2008). Which cortical areas are involved in the PNR is not entirely clear; however, there are projections from the frontal eye fields (FEF) and parietal cortex to the EWN that are involved in vergence movements (Gamlin, 2002). Because of the strong association between vergence and the PNR, and the central role of the EWN in pupil constriction, it is possible that these projections also play a role in the PNR.

### Cognitive influences

To my knowledge, only two studies have directly investigated cognitive influences on the PNR: one published study by Enright (1987); and one of our own studies that we are currently finalizing (Van der Mijn & Mathôt, 2017). The results of these studies are interesting yet puzzling, and more research is certainly needed.

Enright (1987) asked participants to look at two-dimensional drawings of three-dimensional boxes (i.e. drawings that conveyed a sense of perspective). Participants viewed these drawings monocularly, with one eye covered. Participants fixated either on the corner of the box that appeared nearby, or on the corner that appeared far away. Even though the drawing was in a single plane, participants nevertheless made vergence movements as if they looked at the nearby or far-away corner of a real three-dimensional box. But there were no corresponding pupil-size changes: the pupil was not smaller when participants looked at the nearby corner, indicating that vergence was not accompanied by a PNR.

However, the results were very different when participants viewed so-called Neckercubes. A Neckercube is an ambiguous box-like drawing, in which the same corner can be perceived as either nearby or far away. Participants looked at this corner, and indicated whether they subjectively perceived it as the nearby or far-away corner. The results for vergence movements were more-or-less the same as for the regular box drawings (although slightly weaker), but vergence was now accompanied by exceptionally large pupil responses: the pupil was smaller when the corner was subjectively nearby as compared to when it was subjectively far away. However, while the direction of the effect was consistent with a PNR, the size of the effect was unrealistically large, casting doubt on whether it truly was a PNR, or rather an artifact of some unrecognized confound.

In one of our own experiments (Van der Mijn & Mathôt, 2017), we used a setup consisting of three separate displays: a central fixation display at an intermediate distance, a display on the left that was nearby, and a display on the right that was far away (or vice versa). Participants either covertly attended to the nearby or far-away display (while keeping gaze on the central display), or, in a different condition, made an eye movement to the nearby or far-away display. In the covert-attention condition, we found that pupil size did not change as a function of whether participants covertly attended to the nearby or far-away display; this suggests that, unlike the PLR (e.g. Binda et al., 2013a; Mathôt et al., 2013), the PNR is not modulated by covert shifts of attention. In the eye-movement condition, we found that the pupil responded to the distance of the to-be-looked-at display, and, importantly, did so with an extremely low latency; this is reminiscent of the effect of eye-movement preparation on the PLR (Ebitz et al., 2014; Mathôt et al., 2015b). However, these results should be interpreted with caution, because distance is related to other factors that might also affect pupil size, such as brightness and size (i.e. from a retinal point of view, nearby things are generally brighter and larger than far-away things).

In conclusion, it is unclear whether the PNR is affected by cognitive influences. Results from the, as far as I know, only two studies that have investigated this issue suggest that, if cognitive influences on the PNR exist, they are likely smaller than similar influences on the PLR (Enright, 1987; Van der Mijn & Mathôt, 2017).

### Function

The main function of the PNR is likely to increase depth of field for near vision. As described in the section on the PLR, you can see sharply across a wider range of distances with a small pupil than you can with a large pupil (Campbell, 1957; Charman & Whitefoot, 1977). The reason that a large depth of field is especially useful for near vision, is that depth of field is much smaller for nearby than far-away objects; that is, you can simultaneously and sharply see two objects at ten and eleven meters distance, but you cannot simultaneously and sharply see two objects at half and one-and-a-half meters distance.

## The psychosensory pupil response

### Profile

“Man may either blush or turn pale when emotionally agitated, but his pupils always dilate.” (Loewenfeld, 1958, p. 237)

**Figure 6:**
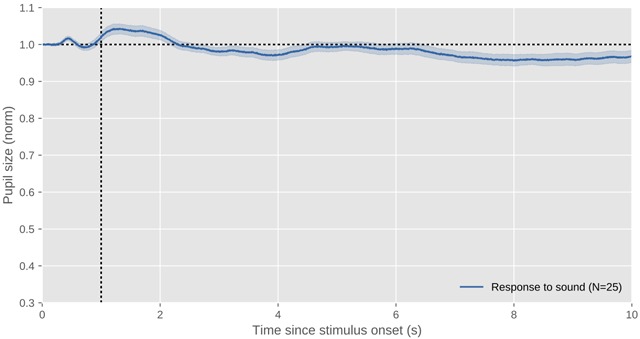
The profile of a typical psychosensory response to sound. This figure shows data of myself while hearing a 1 s burst of auditory white noise. Because the effect is small, this figure is based on more data than the previous figures (N = 25 trials). The x axis indicates time since stimulus onset. The y axis indicates pupil size as a proportion of pre-stimulus pupil size, and is intentionally kept identical to the other figures to illustrate the size of the effect. Errors bars reflect the standard error. All data shown in this figure and others is available through the URL provided at the end of the article.

**Figure 7:**
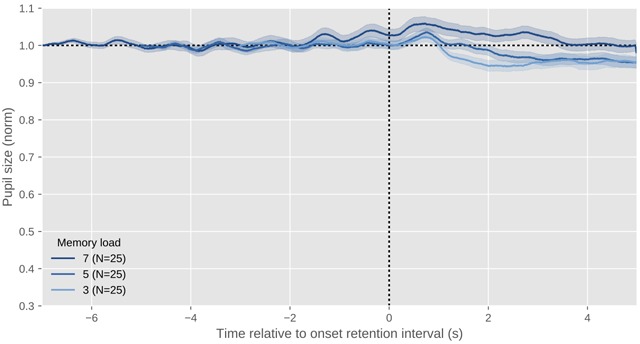
The profile of a typical psychosensory response during working-memory maintenance. This figure shows data of myself while I listen to 3, 5, or 7 digits (N = 25 trials for each set size) played back through a set of desktop speakers, followed by a 5 s retention interval during which I’m keeping the digits in working memory for later recall (recall phase not shown). The x axis indicates time since the onset of the retention interval. The y axis indicates pupil size as a proportion of pre-stimulus pupil size, and is intentionally kept identical to the other figures to illustrate the size of the effect. Errors bars reflect the standard error. All data shown in this figure and others is available through the URL provided at the end of the article.

The pupil dilates after an arousing stimulus, thought, or emotion. Here I call this the *psychosensory pupil response* (PPR), because this term aptly indicates that this response is driven by both sensory and psychological stimuli. But the same response is sometimes also referred to as *reflex dilation, arousal-related dilation*, or *effort-related dilation*. The profile of the PPR varies depending on how it is elicited, but the example data, again from myself, in Figure 6 shows a typical PPR elicited by a sound (a brief burst of auditory noise). Figure 7 shows a typical PPR during a working-memory task (cf. Kahneman & Beatty, 1966).

### Types of psychosensory pupil responses

After decades of intensive study on the PPR, Loewenfeld’s quote from 1958 is still a fair summary of the state of the art: Anything that somehow activates the mind, or anything that increases the mind’s ‘processing load’ (Beatty, 1982), also causes the pupil to dilate (reviewed in Beatty & Lucero-Wagoner, 2000; Einhäuser, 2017; Goldwater, 1972; Laeng, Sirois, & Gredebäck, 2012). Attempts to link specific cognitive processes to the PPR (not mediated by processing load) are therefore, in my view, doomed to fail. However, a useful distinction can be made between an orienting response, which is a brief pupil dilation that is elicited rapidly and involuntarily after something has captured attention (as in Figure 6), and slower arousal- or mental-effort-related responses, which are linked to high-level cognition (as in Figure 7).

#### Orienting response

Sudden events (sounds, movements, painful touch, etc.) trigger an orienting response (Lynn, 1966; Sokolov, 1963): Your head turns and your eyes move toward the source of the event; your muscles tense, you start to sweat, your heart slows down, and your pupils dilate; more generally, your body prepares for whatever may happen next. The orienting response is most pronounced for stimuli that are unexpected (Friedman, Hakerem, Sutton, & Fleiss, 1973) and salient (C. Wang & Munoz, 2014; C. Wang, Boehnke, Itti, & Munoz, 2014); in other words, the orienting response is sensitive to signals, such as novelty and saliency, that indicate whether or not something may be important.

The slight pupil dilation shown in Figure 6 is (part of) an orienting response. It is small compared to the large changes in pupil size induced by the pupil light response (PLR) and the pupil near response (PNR)—or at least it is when triggered by the relatively mild stimuli that are generally used in psychological studies. In humans, the pupil orienting response is characterized by a fast pupil dilation that peaks between 0.5 and 1 s after stimulus onset and dissipates quickly (see also Mathôt, Dalmaijer, Grainger, & Van der Stigchel, 2014b; C. Wang & Munoz, 2014). This orienting response is sometimes followed by a second period of dilation, also visible between 1 and 2 s in Figure 6, which is presumably related to arousal, as discussed below.

#### Mental effort and arousal

In a series of seminal studies, Hess and Polt (1960, 1964) and later Kahneman & Beatty (1966) showed that pupil size is a reliable indicator of mental effort and arousal. This had already been known for a long time, but these studies from the 60s and 70s brought pupillometry to the attention of psychologists (for historic reviews, see Beatty & Lucero-Wagoner, 2000; Loewenfeld, 1958). This type of PPR is endogenous (i.e. generated by an internal cognitive event) and its size and profile are therefore highly variable, presumably reflecting how mental effort and arousal evolve over time (e.g. Mathôt, Siebold, Donk, & Vitu, 2015a; Reimer et al., 2014).

In one study, Hess & Polt (1964) had participants perform mental calculations of different levels of difficulty; for example, 7 × 8 would be easy, whereas 16 × 23 would be difficult. They observed that pupil size reflected the difficulty of the calculation: the harder the calculation, the larger the pupil. This finding was later replicated by Ahern & Beatty (1979) who additionally observed that people with low scores on scholastic aptitude tests showed stronger pupil dilation in response to complex problems than did people with high test scores; presumably, this reflects that low-scoring people needed to invest more effort.

Along the same lines, Kahneman & Beatty (1966) had participants memorize digits, and varied the number of to-be-remembered digits. In line with Hess & Polt (1960), they observed that pupil size reflected the number of digits that were memorized (see also Figure 7). Taken together, these studies, as well as many others that have been conducted since (reviewed in Beatty & Lucero-Wagoner, 2000), showed that pupil size reflects mental effort, cognitive load, or cognitive intensity. Many different terms have been used, but the general finding is clear: whatever activates the mind causes the pupil to dilate.

Hess & Polt (1960) reported a related finding for arousal. They had participants look at images that varied in how arousing they were, and whom the images were arousing to (based on the authors’ subjective impression of the images). The results were clear: when participants viewed arousing images, their pupils dilated, but only when these images were actually arousing to them; for example, men’s pupils dilated most to images of naked women, whereas women’s pupils dilated most to images of babies and naked men. In a follow-up study, Hess, Seltzer, & Shlien (1965) showed that this effect was even modulated by sexual orientation: gay men’s pupils dilated most to images of naked men, whereas straight men’s pupils dilated most to images of naked women. These studies show that pupil size reflects arousal. Importantly, whether arousal is triggered by something negative or positive makes little or no difference (e.g. Bradley, Miccoli, Escrig, & Lang, 2008); that is, pupil dilation depends on arousal (intense v neutral) rather than valence (positive v negative). Pupil size also correlates with other physiological measures of arousal, such as skin conductance (Bradley et al., 2008). In terms of pupil size, the effects of arousal and mental effort appear to be similar: both activate the mind, and both cause the pupils to dilate.

Since the seminal studies by Hess and Polt (1960, 1964; Hess et al., 1965) and Kahneman & Beatty (1966), whose conclusions by and large still hold, there has been little theoretical development in this area—with the notable exception of the adaptive-gain theory discussed below. But pupillometry has since become a standard tool to measure cognitive processing in a wide variety of situations.

### The adaptive-gain theory

The adaptive-gain theory, proposed first by Aston-Jones & Cohen (2005), is an influential theory that deals primarily with the role of the locus coeruleus (LC) in regulating behavior. Because LC activity correlates strongly with pupil size (Joshi, Li, Kalwani, & Gold, 2016), pupillometry has become a popular tool to test predictions of the adaptive-gain theory (e.g. Gilzenrat, Nieuwenhuis, Jepma, & Cohen, 2010; Jepma & Nieuwenhuis, 2011). In my view, this theory has become one of the more interesting frameworks to think about PPRs.

According to Aston-Jones & Cohen (2005), there are two distinct modes of behavior, which they refer to as *exploitation* and *exploration*. Exploitation refers to a behavioral mode in which you are engaged in a single task, such as eating or reading a book; in this mode, you are ‘exploiting’ the rewards that a task has to offer, such as food or the pleasure of reading. Exploitation is associated with intermediate, phasic (bursty) LC activity, and, consequently, an intermediate pupil size. In contrast, exploration refers to a mode in which you are easily distracted and tend to switch from one task to another; in this mode, you are ‘exploring’ different tasks, to find the one that offers the highest reward. Exploration is associated with high, tonic (sustained) LC activity, and, consequently, large pupils. (Low LC activity and small pupils are associated with sleepiness.)

According to the adaptive-gain theory, animals alternate exploitation and exploration to optimize reward. The idea is simple: imagine that you are hungry and open a bag of chips. Initially, while you are still hungry, eating the chips offers a high reward, and you therefore continue to ‘exploit’ this activity. But after some time, you are no longer hungry, and eating chips is no longer rewarding; at this point you switch to exploration, and look for something that offers a higher reward than eating chips. For example, you may start browsing Facebook. It is initially rewarding to see what all your friends have been up to, and you therefore ‘exploit’ Facebook for a while. But once you’ve seen all the new posts (and assuming you don’t suffer from social-media addiction), reward declines, and you again switch to exploration to find a new, more rewarding activity. And so the exploitation-exploration cycle continues.

The adaptive-gain theory has a high face validity: it makes a lot of sense that animals should alternate between exploration and exploitation; in a way it is not even a theory, but rather a description of behavior that animals clearly exhibit. However, the claim that you can track these switches in behavior mode using pupillometry is new and fascinating. In my view, evidence for this claim is still preliminary, but the results of several studies are certainly suggestive.

In an experiment by Jepma & Nieuwenhuis (2011), participants performed the Four Armed Bandit task, a variation of the Iowa Gambling Task. In this task, participants freely select a card from one of four possible decks. Each deck is associated with a particular pay-off, and this pay-off changes gradually over time (rather than instantly, as in the Iowa Gambling Task). This task naturally leads to exploitation-exploration cycles in behavior. Once participants discover that a particular deck has a high pay-off, they will keep selecting cards from this deck: exploitation. However, because pay-offs fluctuate gradually, there comes a point at which the once-profitable deck is no longer profitable, and participants consequently start trying other decks: exploration. The crucial finding by Jepma & Nieuwenhuis (2011) was that pupils were larger during exploration (trying out new decks) than during exploitation (sticking to a profitable deck), as predicted by the adaptive-gain theory. The problem with studies of this kind, however elegant, is that they rely on correlations: behavioral mode (exploitation v exploration) is not manipulated experimentally but inferred from behavior, and switches in behavioral mode are generally correlated with other factors, such as an increase in disappointingly low pay-offs in the study by Jepma & Nieuwenhuis (2011).

Jepma & Nieuwenhuis (2011), and many others, looked at average pupil size over longer periods; that is, they found that exploration is accompanied by a large *tonic* (or *baseline*) pupil size. But another series of experiments showed that exploration is also accompanied by reduced *phasic* pupil responses, that is, a reduced transient pupil dilation in response to stimuli (Gilzenrat et al., 2010). In other words, during exploration the pupil is large but less responsive, a pattern that mirrors neural activity of the LC (Aston-Jones & Cohen, 2005).

A fruitful way to think about exploitation and exploration, and their relationship to phasic and tonic pupil responses, is by taking a hierarchical view: all but the most trivial tasks are composed of subtasks that themselves are composed of yet simpler subtasks. Let’s trace the task hierarchy of my current situation, from simple to complex: I’m sitting behind my computer, writing this paragraph. Being focused on the task of writing is exploitation, compared to switching to another program, for example to check my email—which would be exploration. But at a higher level, the task of sitting behind my computer at all is exploitation, compared to leaving my desk to get a cup of coffee, which is brewing as I type this—which would be exploration. And at a yet higher level, the task of being at work is exploitation, compared going home—which would be exploration.

We can apply this hierarchical view also to experiments, such as the AX-Continuous Performance Task, in which participants are instructed to press a key whenever they see the letter sequence ‘AX’ (e.g. Chiew & Braver, 2013). In this context, exploitation would generally refer to being focused on detecting the AX sequence, whereas exploration would generally refer to being less focused and more prone to distraction. But even when participants are focused on detecting the AX sequence—and are therefore engaged in exploitation at this level of description—they still need to occasionally switch from the micro-task of not pressing a key to the micro-task of pressing a key; in other words, detecting the AX sequence and pressing a key is a form of brief (micro-)exploration, accompanied by a brief (phasic) pupil dilation. In other words, sustained (tonic) and brief (phasic) pupil responses may not be qualitatively different, but rather reflect exploration at different levels and time scales.

In summary, the adaptive-gain theory provides a refined description of what PPRs may reflect. Its distinction between exploitation (small pupils) and exploration (large pupils) is similar to the traditional distinction between calmness (small pupils), and arousal and effort (large pupils). But it is more specific, and theoretically more interesting.

### Neural basis

The neural pathway of the PPR corresponds to the dilation pathway shown in Figure 3b. Fast, orienting-response-like pupil dilation is presumably mediated by activity of the intermediate layers of the superior colliculus (iSC) (C. Wang & Munoz, 2014; C. Wang et al., 2014), and possibly by phasic activation of the locus coeruleus (LC) (Nieuwenhuis, De Geus, & Aston-Jones, 2011). Slower, arousal- and mental-efforted-related pupil dilation is presumably mediated by, or at least correlated with, activity in the hypothalamus and the LC (Aston-Jones & Cohen, 2005; Joshi et al., 2016).

### Function

Researchers have displayed a remarkable lack of interest in the question of why the PPR exists (see Chapter 6 from Bombeke, 2017 for an exception): why do pupils dilate in response to such a wide range of stimuli as sounds, mental effort, and arousal? How does this help us to see the world better, if indeed it does? Consequently, unlike the PLR and PNR, the function of the PPR is mostly unknown. Several researchers have even explicitly rejected the idea that the PPR has any function at all, and consider it to be a non-functional side effect of changes in neural activity. For example, Beatty and Lucero-Wagoner (2000, p. 142) state that “these exceedingly tiny pupillary movements are too small to be of visual consequence.”

In my view, such easy dismissal is unsatisfying. Not only can tiny effects confer an evolutionary benefit (recall the above quote by Barlow, 1972), but it is not clear to me that the PPR is necessarily a tiny effect. Of course, in a laboratory, psychologists use weak stimuli, such as the mental calculations and pictures of naked women used by Hess and Polt (Hess & Polt, 1960, 1964). Such stimuli evoke smallish pupillary dilations, although the 20% diameter change observed by Hess & Polt (1960) is far from negligible. And my own example PPRs shown in Figures 6 and 7 are indeed tiny, because I did not feel like subjecting myself to fear of death or extremely loud noise. But an animal that, confronted with a predator, fears for its life surely shows stronger pupil dilation. To my knowledge, how much pupils dilate under extreme conditions is unclear (and perhaps best left unclear for ethical considerations), and it certainly varies strongly from species to species; for example, whereas the surface of the human pupil can change by a factor of about 16, for gekkos it can change by a factor of about 300 (Denton, 1956).

So how could PPRs help visual perception? Stefan Van der Stigchel and I recently proposed an explanation (Mathôt & Van der Stigchel, 2015). As I described in the sections on the PLR and the PNR, large pupils have both advantages, notably increased visual sensitivity, and disadvantages, notably decreased depth of field and visual acuity. Depending on, among other things, the amount of available light, the advantages outweigh the disadvantages, or the other way around. More specifically, in darkness, you need all the visual sensitivity you can get, and your pupils therefore dilate; but in brightness, visual sensitivity is very high (because light is abundant), and the pupils therefore constrict to increase visual acuity and depth of field.

Presumably, the optimal trade-off between visual sensitivity and visual acuity is not fixed, but depends on the situation. More specifically, if you are calmly focused on a task (exploitation, in the terminology of Aston-Jones & Cohen, 2005), visual acuity may be especially important; for example, reading requires fine discrimination, and thus high visual acuity, as do many other tasks. Therefore, in such situations, the optimal trade-off between sensitivity and acuity may shift toward acuity, causing the pupils to constrict. In contrast, if you are aroused and on the look-out for danger and opportunity (exploration, in the terminology of Aston-Jones & Cohen, 2005), visual sensitivity may be especially important; for example, a subtle movement somewhere may indicate danger, and it needs to be detected as quickly as possible. Therefore, in such situations, the trade-off may shift toward sensitivity, causing the pupils to dilate. In this view, the PPR would not be a distinct kind of pupil response (as I’ve depicted it here); rather, it would be yet another way in which more basic pupil responses (i.e. the PLR and PNR) are modulated by high-level cognition.

Currently, there is little evidence for (or against) a functional role of the PPR as I’ve outlined above (and see Mathôt & Van der Stigchel, 2015). This lack of evidence partly results from the practical difficulties of experimentally manipulating pupil size; that is, to go beyond correlational studies, pupil size needs to be experimentally manipulated, without simultaneously manipulating many other things. This is difficult, but not impossible; for example, artificial pupils can be used (cf. Woodhouse, 1975), the pupil can be dilated with eye drops, or pupil size can be manipulated by changing ambient brightness or color (using the fact that red light leads to a more dilated pupil than blue light, cf. Figure 4). All of these approaches have downsides, but in combination they could provide strong support for (or against) a causal link between pupil responses and performance on various perceptual tasks.

In summary, the function of the PPR may be to find the optimal trade-off between visual sensitivity and acuity for a given situation. In some situations, sensitivity may be more important than acuity, and the pupil may therefore dilate; those situations are generally characterized by high levels of arousal, thus explaining the link between arousal and pupil dilation. In other situations, acuity may be more important than sensitivity, and the pupil may therefore constrict; those situations are generally characterized by calmness.

## Spontaneous fluctuations in pupil size

Even when there is no direct external stimulation, the size of the pupil tends to spontaneously change. Such spontaneous pupil-size fluctuations are sometimes called *hippus* or *pupillary unrest*. *Hippus* is generally used to describe periodic fluctuations, whereas *pupillary unrest* is a more general term that refers to any kind of spontaneous fluctuation.

What, if anything, do spontaneous pupil-size fluctuations reflect? Lowenstein, Feinberg, & Loewenfeld (1963) observed that when participants were awake and alert, their pupils were relatively large and stable; in contrast, as participants grew tired, their pupils became progressively smaller and more restless, dilating and constricting in cycles of several seconds to a minute. Lowenstein et al. (1963) speculated that these fluctuations reflected the waxing and waning of arousal that participants experienced as they grew tired. In line with this, Bouma & Baghuis (1971) observed that pupillary unrest was most likely to occur when participants were left to themselves without a specific task, presumably because this induced drowsiness; when participants were given a task, such as mental calculation, spontaneous pupil-size fluctuations mostly disappeared.

More recently, we showed that spontaneous pupil-size fluctuations correlate with changes in eye-movement behavior (Mathôt et al., 2015a; see also Marzouki, Dusaucy, Chanceaux, & Mathôt, 2017). More specifically, we found that people are more likely to look at conspicuous parts of images or videos when their pupils are small, compared to when their pupils are large. In line with Lowenstein et al. (1963), we interpreted this in terms of arousal, or how much effort people invest their behavior: when pupils are small, indicating low arousal, behavior tends to be guided by the environment, and people therefore tend to look at things that are visually conspicuous (e.g. bright lights, sharp corners, etc; see e.g. Theeuwes, Kramer, Hahn, & Irwin, 1998). In contrast, when pupils are large, indicating high arousal, behavior tends to be guided endogenously (by the task), and people therefore tend to look at things that are task-relevant but not necessarily visually conspicuous (e.g. things that look like keys if you’re looking for your keys). This finding supports the notion that spontaneous pupil-size fluctuations track fluctuations in levels of arousal, and further suggest that these fluctuations affect our behavior in subtle ways.

Reimer and colleagues (2014, 2016; see also Joshi et al., 2016) investigated the changes in neural activity that accompany spontaneous pupil-size fluctuations. They directly measured brain activity, using a variety of techniques, as well as pupil size in awake, non-moving mice. First, they observed that mice also exhibit pupillary unrest, albeit in slightly faster cycles than humans. Furthermore, they observed that during constriction, visual brain areas became less responsive (Reimer et al., 2014), suggesting that the mice were less attentive to their environment. They also observed that spontaneous pupil-size fluctuations were correlated with activity in noradrenergic projections to the cortex (Reimer et al., 2016), suggesting that the locus coroeleus (LC), an important source of noradrenergic input to the cortex (Aston-Jones & Cohen, 2005), may be driving pupil-size fluctuations. However, activity in several other brain areas also correlates with pupil size, and this makes it difficult to pinpoint one specific brain area as the main driver of pupil-size fluctuations (Joshi et al., 2016).

Taken together, spontaneous fluctuations in pupil size, often called pupillary unrest or hippus, seem to reflect fluctuations in level of arousal, and are especially pronounced when someone is tired (Lowenstein et al., 1963) and not engaged in a specific task (Bouma & Baghuis, 1971). Like the psychosensory pupil response, spontaneous pupil-size fluctuations are linked to activity in the LC (Joshi et al., 2016; Reimer et al., 2016).

## Summary

The size of the pupil is controlled by two muscles that are located in the iris: the iris sphincter muscle, which is innervated by the parasympathetic nervous system and causes the pupil to constrict; and the iris dilator muscle, which is innervated by the sympathetic nervous system and causes the pupil to dilate (Kardon, 2005; McDougal & Gamlin, 2008).

There are three distinct kinds of pupil responses. The pupil light response is a constriction of the pupil in response to increased brightness. The light response is partly reflexive, but is modulated by many factors related to high-level cognition: visual awareness, covert visual attention, eye-movement preparation, and subjective brightness (Binda & Murray, 2014; Mathôt & Van der Stigchel, 2015).

The pupil near response is a constriction of the pupil that occurs when gaze shifts from a far-away to a nearby object; the near response is generally accompanied by vergence eye movements and lens accommodation, and these three eye movements together are called the near triad (Mays & Gamlin, 1995). The near response is, at least in part, a reflex; whether, like the light response, it can be modulated by high-level cognition is still unclear (Enright, 1987; Van der Mijn & Mathôt, 2017).

Pupil constriction in response to light and near-fixation presumably serves to increase visual acuity and depth of field by decreasing how much of the eye’s lens is exposed; in addition, pupil dilation in darkness may reduce the time required for dark adaptation by reducing (but not eliminating) the difference in retinal illumination when going from a bright to a dark environment (Woodhouse & Campbell, 1975).

The psychosensory pupil response is a dilation of the pupil in response to increased arousal, mental effort—or more generally, anything that ‘activates the mind’ (Beatty & Lucero-Wagoner, 2000). The same response is sometimes also referred to as reflex dilation, arousal-related dilation, or effort-related dilation. The psychosensory response can take the form of a fast-but-transient pupil dilation as part of an orienting response (C. Wang & Munoz, 2015). It can also take the form of a slower, but more prolonged dilation that reflects more sustained increases in arousal or mental effort. Spontaneous fluctuations in pupil size, often called *pupillary unrest* or *hippus*, are in many ways similar to the psychosensory pupil response, with the notable difference that there is no clear external trigger, but rather a spontaneous waxing and waning of arousal (Bouma & Baghuis, 1971; Lowenstein et al., 1963).

The adaptive-gain theory is an influential theory that has linked the psychosensory pupil response to modes of behavior (Aston-Jones & Cohen, 2005): a small pupil would reflect drowsiness; an intermediate-size pupil would reflect *exploitation*, a state in which attention is narrowly focused on a single task; and a large pupil would reflect *exploration*, a distractible mode that is characterized by switching between tasks. In other words, according to the adaptive-gain theory, the psychosensory pupil response reflects a shift from exploitation to exploration. The link between pupil size and behavior mode is supported by several studies (e.g. Gilzenrat et al., 2010; Jepma & Nieuwenhuis, 2011).

Remarkably, the question of *why* the pupil dilates during exploration relative to exploitation has received little interest. One possibility is that large pupils increase visual sensitivity (how well you can detect faint stimuli) at the expense of visual acuity (how well you can distinguish fine detail), and that increased visual sensitivity is especially beneficial during exploration, or more generally, during situations in which arousal is high (Mathôt & Van der Stigchel, 2015).

In conclusion, pupil responses are an important class of eye movements. Like other kinds of eye movements, such as saccades and smooth pursuit, pupil responses have properties of reflexive as well as voluntary action. Although there is still much to learn about the function of pupil responses, they are clearly an important part of active vision.

## Materials

Manuscript materials are available from:

- https://github.com/smathot/pupillometry_review
